# Tissue-Specific Expression of the Chicken *Calpain2* Gene

**DOI:** 10.4061/2010/373241

**Published:** 2010-08-02

**Authors:** Zeng-Rong Zhang, Xiao-Song Jiang, Hua-Rui Du, Xiao-Cheng Li, Qing Zhu, Yi-Ping Liu

**Affiliations:** ^1^Institute of Poultry Sciences, Sichuan Animal Science Academy, Chengdu, Sichuan 610066, China; ^2^Breeding Center, College of Animal Science and Technology, Sichuan Agricultural University, Ya'an, Sichuan 625014, China; ^3^Sichuan Dahen Poultry Breeding Company, Chengdu, Sichuan 610066, China

## Abstract

We quantified chicken *calpain 2* (*CAPN2*) expression in two Chinese chicken breeds (mountainous black-bone chicken breed [MB] and a commercial meat type chicken breed [S01]) to discern the tissue and ontogenic expression pattern and its effect on muscle metabolism. Real-time quantitative PCR assay was developed for accurate measurement of the *CAPN2* mRNA expression in various tissues from chickens of different ages (0, 2, 4, 6, 8, 10, and 12 weeks). Results showed that the breast muscle and leg muscle tissues had the highest expression of *CAPN2* compared to the other tissues from the same individual (*P* < .05). Overall, the *CAPN2* mRNA level exhibited a “rise” developmental change in all tissues. The S01 chicken had a higher expression of the *CAPN2* mRNA in all tissues than the MB chicken. Our results suggest that chicken *CAPN2* expression may be related to chicken breeds and tissues.

## 1. Introduction

Nowadays a highlighted issue for an increasing number of consumers is the problem of meat tenderness, as the result of its physicochemical and biochemical mechanisms acting mainly on myofibrillar structures postmortem. Calpains play an important role in degradation of muscle protein. Because calpastatin inhibits both calpain1 and calpain2, it remains unclear whether one or both calpains are active in postmortem muscle. Most of the studies that have used appropriate methodology [1] have found the same effect of postmortem storage on the activity of the components of the calpain system [2–4]. This conclusion seems to be based on the finding that calpain2 is not autolyzed during postmortem storage [5, 6]. But some studies also have now suggested that both unautolyzed calpain1 and calpain2 are proteolytically active [7, 8].

The lack of expression data of the *calpain2* gene in chicken makes it difficult to verify the role of calpain2 in control of meat quality and carcass traits. In this study, we aimed to (1) develop a convenient approach to quantify the abundance of the *calpain2* transcripts in chicken tissues and (2) determine tissue distribution and ontogenic expression of this gene, particularly in muscle tissues.

## 2. Materials and Methods

### 2.1. Animals

Thirty-six chickens at different ages from two breeds/populations, Mountainous black-bone chicken (MB) and a commercial chicken (S01) from Sichuan province, were used in this study. All populations were raised under the same condition and were randomly selected. Tissue samples (including heart, liver, breast muscle, leg muscle, brain, and abdominal fat) from MB chickens were collected at 0, 2, 4, 6, 8, and 10 w, respectively; we slaughtered four chickens at each time point. We also collected tissue samples from six S01 chickens at 10 w and six MB chickens at 12 w. Tissue samples were quick-frozen in liquid nitrogen and then stored at −80°C for total RNA extraction.

### 2.2. RNA Isolation and cDNA Synthesis

Total RNA was isolated from the heart, liver, brain, breast muscle, and leg muscle tissues by using the TRIzol reagent (Invitrogen). The quality of RNA was determined by the A_260/280_  absorbance ratio (1.6–1.8) and the integrity of the 18S and 28S rRNA bands on 1% formaldehyde agarose gel. Isolated RNAs were treated with 8 *μ*L DNase (Fermentas) for 20 minutes at 37°C and stored at −80°C. The cDNA was synthesized using the ImProm-II Reverse Transcription System (TaKARa) according to the manufacturer's instructions. The reaction was performed in a volume of 10 *μ*L containing 5×PrimerScript Buffer, 10 mM of each dNTPs, 40 U/*μ*L RNase Inhibitor, and 2.5 *μ*M oligo-dT Primer. The reverse transcription was maintained at 30°C for 10 minutes, 45°C for 25 minutes, 99°C for 5 minutes, and ended with a 4°C for 5 minutes, then stored at −20°C.

### 2.3. Real-Time Quantitative PCR (RT-qPCR) Assay for the CAPN2 Gene

According to the chicken *CAPN2 *mRNA sequence in GenBank (accession number NM_205080.1), a pair of primers were designed by using Oligo 6.0 (Table 1). The expression levels of chicken *CAPN2* gene were detected by using the SYBR Green I assay on an IQ5 real-time PCR thermal cycle instrument (Bio-Rad, German) and were normalized to the expression of the *β-actin *gene (*ACTB*; GenBank Accession No. AF047874). Relative transcript quantification was performed using standard curves generated for the *ACTB *and *CAPN2 *genes based on a 7-fold serial dilution of the pooled cDNA product prepared from a subset of the gastrocnemius samples. The cycling conditions consisted of an initial single cycle of 95°C for 3 minutes, followed by 35 cycles of 15 s at 94°C and 1 minute at 60°C. Reactions were performed in a volume of 25 *μ*L and included 2.0 *μ*L cDNA template, 1.0 *μ*L of each specific primer (Table 1), 12.5 *μ*L SYBR Green PCR Master Mix (TaKaRa, Japan), 1.0 *μ*L calibration liquid (BIO-RAD), 7.5 *μ*L PCR-grade water, and 1.0 *μ*L cDNA. All real-time quantitative PCR amplifications were performed in triplicate for each sample and were analyzed using the 2^−ΔΔCt^ method previously described.

### 2.4. Statistical Analysis

Expression data were described as (least square mean ± standard error) and were analyzed using the SAS 8.0 for Windows Software (SAS Institute Inc., Cary, NC). The expression levels of the *CAPN2* gene between the tissue and age-related samples of the same breed and those samples from the two breeds with the same age were analyzed by the one-way ANOVA and Student's *t*-test, respectively. *P* value < .05 was considered as statistically significant.

## 3. Results

### 3.1. Validation of the RT-qPCR Assays for the CAPN2 Gene

Relative mRNA quantification was performed using standard curves generated for the *ACTB *and *CAPN2 *genes based on a serial dilution of cDNA. In the current assay, the amplification efficiency of the *ACTB *and *CAPN2 *genes were 91.9% and 92.60%, respectively, which were approximately within the expected theoretical values. 

### 3.2. Tissue Distribution of the CAPN2 mRNA

Quantitative PCR analysis showed that the *CAPN2* gene was expressed in all six MB chicken tissues. The *CAPN2 *gene transcript had the highest expression level in breast muscle and leg muscle whereas it had the lowest expression level in liver tissue. The mRNA levels of the *CAPN2* gene in breast muscle and leg muscle were higher than those in other tissues from the same chicken (*P* < .05) except at 10 w (Table 2).

### 3.3. Ontogenic Expression of the CAPN2 mRNA

**Figure 1:**
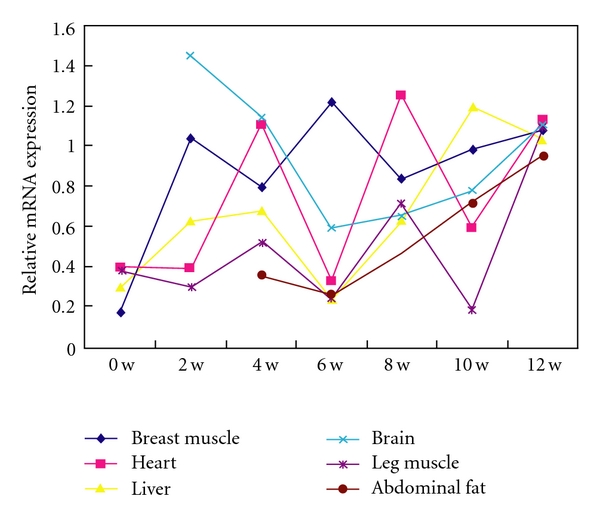
Ontogenic expression of the *CAPN2* mRNA in tissues collected from chickens at different ages. The values were normalized to the endogenous *ACTB *expression in the same tissue, and the expression level of the *CAPN2* gene in different tissues at 12 w was arbitrarily set to 1.0.

We analyzed the developmental changes of the *CAPN2* mRNA expression for each tissue in MB chickens with different ages. As shown in Figure 1, the *CAPN2 *mRNA in breast muscle had the highest expression at 6 w and the lowest expression at birth. In leg muscle tissue, the highest expression of the* CAPN2 *gene was at 12 w and the lowest expression at birth. Overall, the *CAPN2* mRNA levels exhibited a rise developmental change in all tissue.

### 3.4. Comparison of the CAPN2 Gene Expression between the MB and S01 Chickens

**Figure 2:**
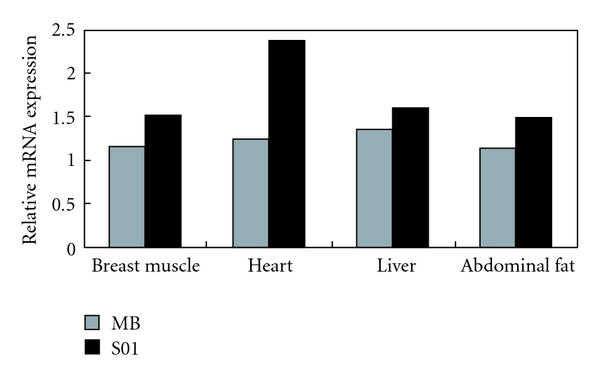
The relative mRNA expression of the *CAPN2* gene in tissues collected from chickens belonging to the meat-type S01 and the mountainous black-bone chicken (MB) breeds at age of 10 weeks.

To characterize whether the expression of the *CAPN2* gene had a breed-specific feature, we analyzed the expression levels of this gene in two chicken breeds.  Figure 2 presents the expression pattern of the *CAPN2 *mRNA in the MB and S01 chickens at 10 w. The meat-type S01 chicken had a higher expression of the *CAPN2* gene than that of MB chicken in all tissues.

## 4. Discussion

Although previous studies on calpain protease activity indicate that the 2 calpains, calpain1 and calpain2, have an important role in the postmortem proteolysis that increases meat tenderness, it remains unclear how the calpains function in postmortem muscle.

In birds, and in particular the chicken breast muscle, the role of postmortem proteolysis is poorly documented, and the few studies performed did not take into account the particularities of the calcium-dependent proteases in these species [9–12].

In this study, we quantified the chicken *CAPN2 *gene tissue distribution and ontogenic expression. The *CAPN2 *mRNA was expressed in all six different tissues studied in the current study, with a dominant expression in breast muscle and leg muscle tissues. The overall pattern of the *CAPN2 *expression in breast muscle was different from that of leg muscle. It is well known that the breast muscle of chicken is made of white fast oxidative glycolytic fibers that whereas the leg muscle has slow oxidative red aerobic fibers [9]. We also found the relative expression level of the* CAPN2* mRNA in the S01 chicken was higher than that in the MB chicken. Investigation of the role of the calpain 2 and its muscle protein substrates in these two chicken breeds may further explain the observed variation in meat tenderness. These results are necessary for knowing the effects of *CAPN2* on the regulation of muscle protein metabolism and for defining the biological significance of degradation of the myofibrillar proteins in chicken, as well as potential applications in marker-assisted selection in chicken breeding. Regarding to experiment error, the *CAPN2* mRNA quantification data was reliable.

## 5. Conclusions

In conclusion, we have developed a highly sensitive real-time PCR method to detect tissue distribution and ontogenic expression of the* CAPN2* mRNA in chicken. We found that the *CAPN2* expression may be related with tissue in chicken. Future studies will be essential to determine the biochemical character of the muscles from the two chicken breeds and to discern the factors that contribute to differences in their meat quality, as well as to define the role of the calpain 2.

## Figures and Tables

**Table 1:** Primers of real-time PCR the chicken *β* − *actin* and *CAPN2* gene.

Gene	Primer sequence (5'–3')	*T*m (°C)
**β*-actin*	F: GCCAACAGAGAGAAGATGACAC	60
	R: GTAACACCATCACCAGAGTCCA	
*CAPN2*	F: TAGACCTACGGAGCTGTGTTCCT	60
	R: TTCAGAGTGAGGGAGGCAATAG	

*T*m: annealing temperature.

**Table 2:** Comparison of the tissue distribution of CAPN2 mRNA at different growth points.

Growth point	Breast muscle	Heart	Liver	Brain	Leg muscle	Abdominal fat
0 w	15.60^b^ ± 2.58	5.20^c^ ± 2.58	1.08^c^ ± 0.74	∖	25.22^a^ ± 2.58	∖
2 w	10.52^a^ ± 1.03	2.28^b^ ± 0.15	1.03^b^ ± 0.03	6.34^b^ ± 1.02	9.15^a^ ± 0.90	
4 w	8.12^ab^ ± 2.33	6.39^b^ ± 2.13	1.07^b^ ± 0.62	4.67^b^ ± 0.53	15.06^a^ ± 4.07	4.03^b^ ± 1.97
6 w	19.30^a^ ± 3.35	5.11^b^ ± 0.20	1.03^b^ ± 0.07	7.39^b^ ± 0.48	18.79^a^ ± 2.04	7.97^b^ ± 0.54
8 w	19.95^a^ ± 1.97	7.65^b^ ± 1.65	1.13^b^ ± 0.57	5.23^b^ ± 1.01	21.90^a^ ± 7.58	6.67^b^ ± 2.06
10 w	4.16 ± 2.91	2.46 ± 0.64	1.36 ± 0.07	4.01 ± 0.26	3.83 ± 2.92	3.35 ± 2.24
12 w	6.06^ab^ ± 3.77	4.40^ab^ ± 1.56	1.09^b^ ± 0.36	3.70^ab^ ± 0.75	11.85^a^ ± 2.65	2.36^b^ ± 0.59

Note. Values in a line without a common lowercase mean that the relative quantities of the *CAPN2 *gene differ greatly significantly at the same age (P <0.05). Expression data were based on the mountainous black-bone chicken (MB). For each growth point, we used the Ct value of the liver as the control to calculate the expression values of the *CAPN2 *mRNA in other tissues.
